# Correction: Facile synthesis of an α-Fe_2_O_3_–TiO_2_-MXene heterojunction for enhanced acetone gas sensors

**DOI:** 10.1039/d5ra90017d

**Published:** 2025-02-25

**Authors:** Zhenyuan Yang, Ying Chen

**Affiliations:** a School of Science, Hubei University of Technology Wuhan 430068 China chenying@hbut.edu.cn; b Hubei Engineering Technology Research Center of Energy Photoelectric Device and System, Hubei University of Technology Wuhan 430068 China

## Abstract

Correction for ‘Facile synthesis of an α-Fe_2_O_3_–TiO_2_-MXene heterojunction for enhanced acetone gas sensors’ by Zhenyuan Yang *et al.*, *RSC Adv.*, 2025, **15**, 3040–3046, https://doi.org/10.1039/D4RA08407A.

The authors regret that an incorrect version of Fig. 6 was included in the original article. The correct version of Fig. 6 is presented below.

**Fig. 6:**
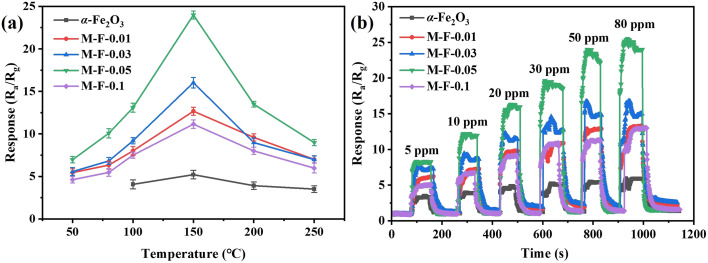
(a) Response values of α-Fe_2_O_3_, M-F-0.01, M-F-0.03, M-F-0.05, and M-F-0.1 to 50 ppm acetone at different temperatures. The error bar is from the mean values and standard deviations of three tested groups of gas sensors under the same experimental conditions. (b) Dynamic response values to different concentrations of acetone at 150 °C.

The Royal Society of Chemistry apologises for these errors and any consequent inconvenience to authors and readers.

